# Appropriateness of the institute certification system for esophageal surgeries by the Japan Esophageal Society: evaluation of survival outcomes using data from the National Database of Hospital-Based Cancer Registries in Japan

**DOI:** 10.1007/s10388-018-0646-4

**Published:** 2018-10-15

**Authors:** Satoru Motoyama, Eri Maeda, Masahiko Yano, Takushi Yasuda, Masaichi Ohira, Yuichiro Doki, Yasushi Toh, Takahiro Higashi, Hisahiro Matsubara, The Japan Esophageal Society

**Affiliations:** 1 The Japan Esophageal Society, Tokyo, Japan; 2 Esophageal Surgery, Akita University Hospital/Comprehensive Cancer Control, Akita University Graduate School of Medicine, 1-1-1 Hondo, Akita, 010-8543 Japan; 3 Department of Environmental Health Sciences, Akita University Graduate School of Medicine, Akita, Japan; 4 Center for Cancer Registries and Division of Health Services Research, Center for Cancer Control and Information Services, National Cancer Center, Tokyo, Japan

**Keywords:** Esophageal cancer, Esophagectomy, Institute certification, Survival outcome

## Abstract

**Background:**

Since 2013, The Japan Esophageal Society has been certifying “Authorized Institute for Board Certified Esophageal Surgeon (AIBCES)” to contribute to improving national medical care by enhancing the professional knowledge and skills of esophageal surgeons. However, the appropriateness on this certification system has not yet been verified. Our aim was to assess the appropriateness of the institute certification system for esophageal surgeries used by the medical society.

**Methods:**

Using data from the National Database of Hospital-based Cancer Registries, we analyzed the 5-year overall survival rates among 2135 patients with thoracic esophageal cancer who underwent an esophagectomy at 53 AIBCES or 141 non-AIBCES.

**Results:**

There were 1343 (63%) patients who underwent surgery at an AIBCES and 792 (37%) who underwent surgery at a non-AIBCES. Registered patients were followed up for a median of 53 (range 1–88) months. Over the followed-up period examined, 670 (50%) patients treated at an AIBCES died and 455 (57%) treated at a non-AIBCES died. Comparison of the Kaplan–Meier survival curves indicated that patients with cStage II or cStage III disease treated at an AIBCES had significantly better 5-year survival rates than those treated at a non-AIBCES (55.4% vs. 44.9% and 38.0% vs. 30.3%, respectively). Univariate and multivariate analyses stratified based on stages and adjuvant therapies revealed that institute certification (AIBCES vs. non-AIBCES) is a significant independent factor for 5-year survival.

**Conclusions:**

The institute certification system used by the Japan Esophageal Society may be appropriate, as indicated by improved 5-year survival outcomes. The institute certification system has the potential to contribute to a more appropriate medical delivery system in the future.

## Introduction

Thoracic esophageal cancer is an aggressive cancer characterized by rapid and extensive progression and a poor prognosis [1, 2]. The surgery for this disease is a complex procedure that requires extensive lymph node dissection, from the neck to the abdomen, and reconstruction over a long distance in the mediastinum using stomach or colon/jejunum [1, 3]. Moreover, the preoperative chemotherapy and/or radiotherapy usually administered in cases of Stage II–IVa esophageal cancer in an effort to improve survival outcome induces additional surgical stress and complications in the patient [4, 5]. Consequently, esophagectomy for esophageal cancer requires a surgeon with experience in the relevant surgical techniques and a well-trained staff administering perioperative care.

Since 2013, the Japan Esophageal Society has been certifying “Authorized Institute for Board Certified Esophageal Surgeon” (AIBCES) to contribute to improving national medical care by enhancing the professional knowledge and skills of esophageal surgeons. It is mandated that AIBCES are facilities that provide surgeons with education and training toward becoming a board-certified esophageal surgeon. Although many board-certified esophageal surgeons have emerged through training at an AIBCES, the Japan Esophageal Society has not yet verified the appropriateness on this certification system.

The purpose of the present study is to verify the appropriateness of the certification system for AIBCES by the Japan Esophageal Society. To do so, we used the National Database of Hospital-based Cancer Registries to examine survival outcomes among patients with thoracic esophageal cancer who underwent an esophagectomy at an AIBCES or a non-AIBCES.

## Patients and methods

### Authorized Institute for Board Certified Esophageal Surgeon

The Japan Esophageal Society began certifying AIBCES in 2013. The provisions for certification include (1) more than 100 patients diagnosed or treated in hospital and more than 50 surgeries for esophageal disease within a period of 5 years, (2) employment of full-time, board-certified esophageal surgeons, (3) presence of a training curriculum for board-certified esophageal surgeons, (4) radiation therapy is fully established, (5) autopsy and rapid pathological diagnosis are fully established, (6) continuous performance of education (case conferences, death conferences, and so on) in esophagology, (7) performance of relevant research (report articles, presentations at the annual meeting of the Japan Esophageal Society), and (8) contribution to the Comprehensive Registry in Esophageal Cancer in Japan. First, selection of AIBCES was deliberated in 2012 and delivered in 2013. This means that AIBCES certified in 2013 were selected based on research and clinical achievements during 2007–2011, which included the patients treated in 2008. Sixty-eight facilities were certified in 2013. Another 45 facilities were then certified in 2014 based on research and clinical achievements during 2008–2012, which also included the patients treated in 2008.

### National Database of Hospital-based Cancer Registries

We retrieved 2008 data from the National Database of Hospital-based Cancer Registries along with 5-year survival information from the National Cancer Center, Tokyo, Japan [6]. There were 428,195 cases of cancer listed in the 2008 Hospital-based Cancer Registry. The registry data include the following information on individual cancer patients: (1) clinical profiles, including birth date, sex, tumor topology, and histology code according to the International Classification of Disease for Oncology, third edition (ICD-O-3); (2) clinical and pathological tumor–node–metastasis (TNM 6th Edition) stage based on the Union for International Cancer Control (UICC); (3) diagnosis date; (4) first-line treatment details; and (5) survival information (follow-up time after diagnosis of cancer). We extracted the data for patients diagnosed with thoracic esophageal cancer (C151, C153–155) and treated surgically at a registered hospital for thoracic esophageal cancer, and for patients diagnosed at another hospital but treated surgically at a registered hospital for thoracic esophageal cancer.

### Analysis and statistical methods

We divided the patients into two groups, those who underwent surgery at an AIBCES (certified in 2013 or 2014) or a non–AIBCES and analyzed the patient backgrounds and 5-year survival rates in the two groups.

Statistical comparisons between patients in the AIBCES and non-AIBCES groups were carried out using Student’s *t* test, the Chi squared test, Fisher’s exact test, or the Wilcoxon-type test for trend, depending on the type and distribution of the variables. Overall survival was characterized using Kaplan–Meier plots. Survival curves were compared between the two groups using log-rank tests. Unadjusted Cox proportional hazards models were used to estimate the hazard ratios for the treatment at AIBCES, along with demographic, oncologic, and adjuvant therapy factors. Incidence rate ratios were calculated for T classification (T3–4 vs. Tis-2), N classification (N1–3 vs. N0), clinical stages, and adjuvant therapies because these variables failed to meet the assumption of proportionality. A multivariate Cox proportional hazards regression model was developed to evaluate the effect of treatment at an AIBCES on survival after adjusting for age, sex, stratification of clinical stages, and adjuvant therapies. Stratification of clinical stages and adjuvant treatments was used to control for the effects of these variables without assuming proportional hazards. A two-sided value of *p* = 0.05 was set to define statistical significance. We performed all statistical operations using Stata 14-MP (StataCorp LP, College Station, TX, USA).

## Results

The Hospital-based Cancer Registry for 2008 listed 9380 patients registered with esophageal cancer (C15, including cervical esophageal cancer [C150] and abdominal esophageal cancer [C152]). Selected for analysis were patients at 208 hospitals able to provide 5-year survival data for all cancers for more than 90% of their patients. Among a total of 7360 patients, 2135 (29%) underwent esophagectomy for thoracic esophageal cancer.

The study participants were treated surgically at 53 AIBCES and 141 non-AIBCES (Table 1). Their mean age( ± SD)was 65.1 ± 8.2 years and included 1826 (86%) males. There were 8 (< 1%) patients with cTis tumors, 636 (30%) with cT1 tumors, 455 (21%) with cT2 tumors, 867 (41%) with cT3 tumors, and 100 (5%) with cT4 tumors. Lymph node metastasis was detected in 1981 (51%) patients, but not in the remaining 995 (47%) patients. Distant metastasis (M1) was detected in 163 (8%) patients, but not in 1914 (90%) patients. Nine patients had cStage 0 disease, 507 (24%) had cStage I, 750 (35%) had cStage II, 636 (30%) had cStage III, and 167 (8%) had cStage IV. Transthoracic esophagectomies under thoracotomy were performed in 1762 (83%) patients, and thoracoscopic esophagectomies were done in 364 (17%) patients. One hundred and forty-six (49%) patients were treated with surgery only, while 1087 (51%) were treated with surgery plus adjuvant or neoadjuvant chemotherapy, chemoradiotherapy, or radiotherapy.

**Table 1:** Comparison of patient characteristics

	AIBCES	Non-AIBCES	*p*
Number of institutes	53	141	
Number of patients	1343	792	
Age (mean, SD)	64.7 (8.2)	65.8 (8.2)	<0.01^1)^
Number of males (%)	1146 (85%)	680 (85%)	0.74^2)^
T classification			0.55^3)^
Tis	8 (1%)	0 (0%)	
T1	424 (32%)	212 (27%)	
T2	240 (17%)	215 (27%)	
T3	578 (43%)	289 (37%)	
T4	56 (4%)	44 (6%)	
T*x*	37 (3%)	32 (3%)	
N classification			0.34^3)^
N0	609 (45%)	386 (49%)	
N1	675 (50%)	366 (46%)	
N2	18 (1%)	16 (2%)	
N3	3 (0%)	3 (0%)	
N*x*	38 (3%)	21 (3%)	
N classification			0.32^2)^
N0	609 (45%)	386 (49%)	
N1–3	696 (52%)	385 (49%)	
N*x*	38 (3%)	21 (3%)	
M classification			0.12^2)^
M0	1190 (89%)	724 (91%)	
M1	112 (8%)	51 (6%)	
Unknown	41 (3%)	17 (2%)	
Clinical stage (UICC)			0.26^3)^
cStage 0	9 (1%)	0 (0%)	
cStage I	325 (24%)	182 (23%)	
cStage II	436 (33%)	314 (40%)	
cStage III	420 (31%)	216 (27%)	
cStage IV	113 (8%)	54 (7%)	
Unknown	40 (3%)	26 (3%)	
Surgery			<0.01^4)^
Thoracotomy	1085 (81%)	677 (86%)	
Thoracoscopic	254 (19%)	110 (14%)	
Unknown	4 (0%)	5 (1%)	
Adjuvant therapy			<0.01^4)^
No radiation or chemotherapy	642 (48%)	404 (51%)	
+Radiation therapy	20 (2%)	21 (3%)	
+Chemotherapy	552 (41%)	245 (31%)	
+Radiation and chemotherapy	127 (10%)	122 (15%)	
Unknown	2 (0%)	0 (0%)	

*AIBCES* Authorized Institute for Board Certified Esophageal Surgeon

Statistical comparisons were made using ^1)^ Student’s *t* test, ^2)^ the Chi squared test, ^3)^ Wilcoxon-type test for trend, ^4)^ Fisher’s exact test

**Fig. 1:**
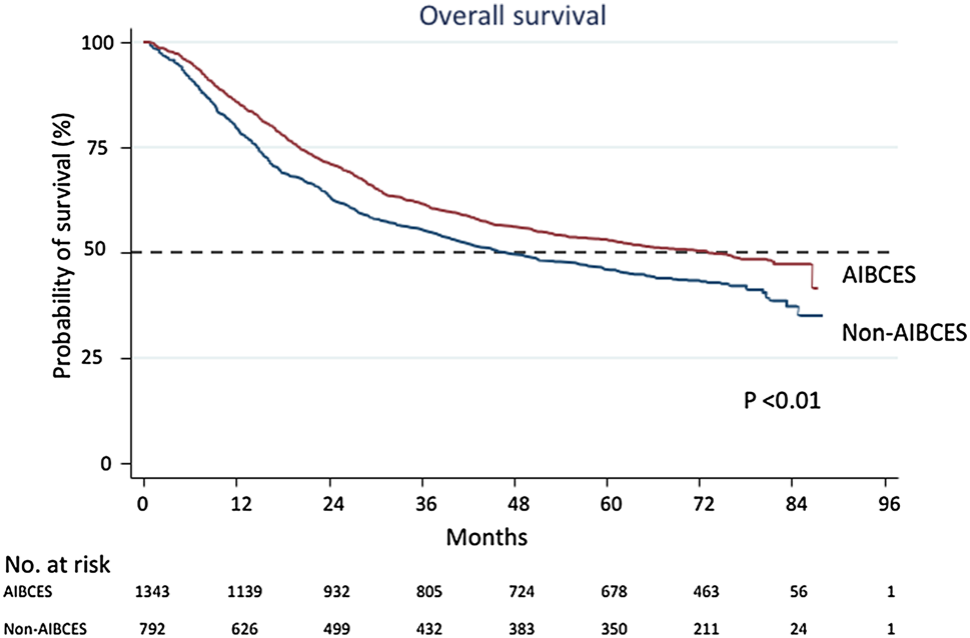
Kaplan–Meier curves for overall survival of patients with all stages thoracic esophageal cancer operated on at Authorized Institutes for Board-Certified Esophageal Surgeons (AIBCES) and non-AIBCES. There is a significant difference in 5-year overall survival

**Fig. 2:**
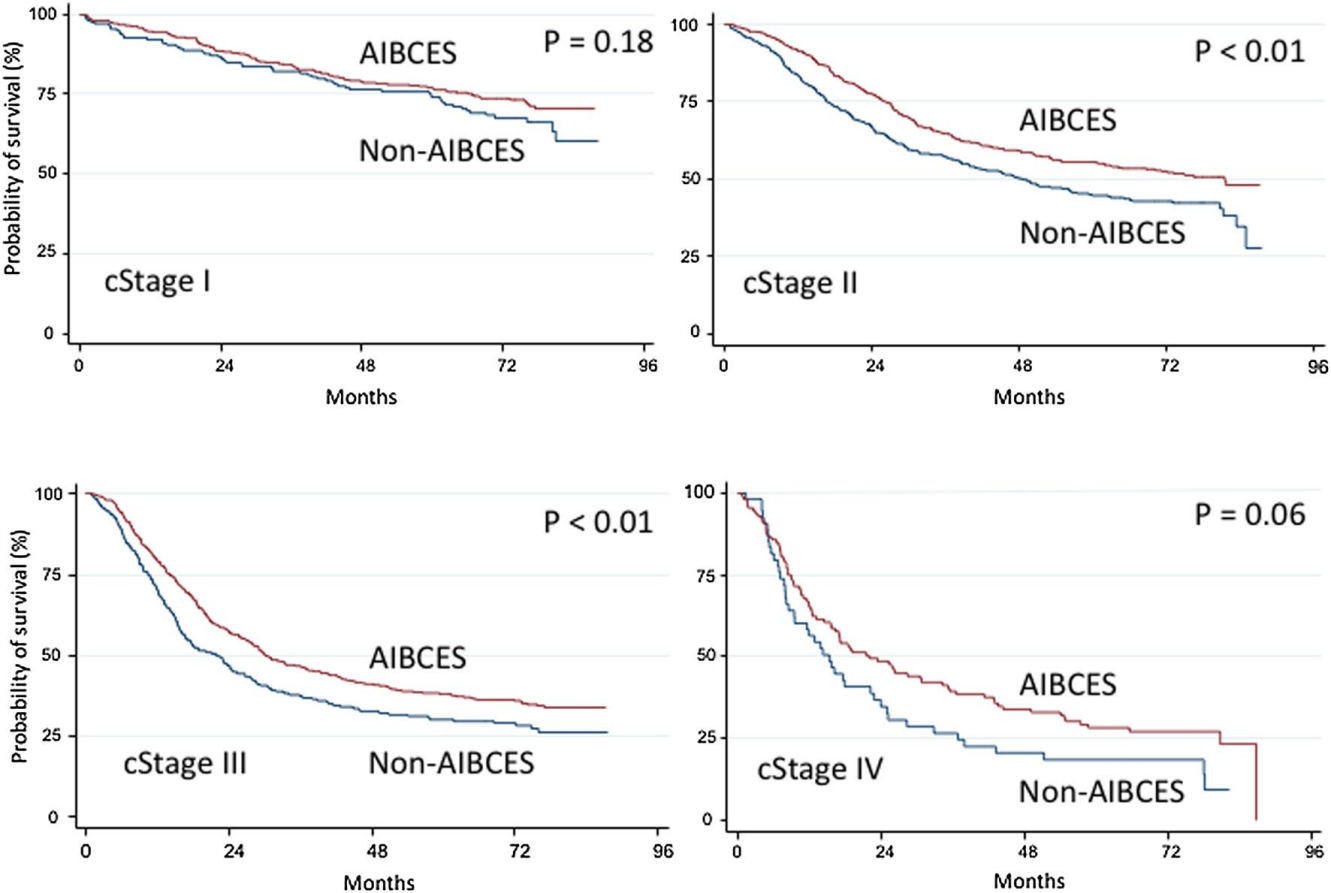
Kaplan–Meier curves for overall survival of patients with cStage I, II, III, or IV thoracic esophageal cancer operated on at Authorized Institutes for Board Certified Esophageal Surgeons (AIBCES) and non-AIBCES. There are significant differences in 5-year overall survival for cStage II and III

There were 1343 (63%) patients who underwent surgery at an AIBCES and 792 (37%) who underwent it at a non-AIBCES. The average numbers of surgeries per year at AIBCES and non-AIBCES were 25.3 and 5.6, respectively. The patient backgrounds differed between two groups with respect to age, surgical approach, and adjuvant therapies (Table 1). Pre- or postoperative chemotherapy was administered to 41% of patients treated at an AIBCES and to 31% treated at a non-AIBCES. Pre- or postoperative chemoradiotherapy was administered at 10% of AIBCES and at 15% of non-AIBCES. Registered patients were followed up for a median of 53 (range 1–81) months. Over the follow-up period examined, 670 (50%) patients treated at an AIBCES died and 455 (57%) treated at a non-AIBCES died (Table 2). Comparison of the Kaplan–Meier survival curves using log-rank tests indicated a significant (*p* < 0.01) difference between the AIBCES and non-AIBCES groups (Fig. 1). There were also significant differences in the 5-year survival rates for patients with cStage II (55.1% vs. 44.9%) and cStage III (38.0% vs. 30.3%) disease between AIBCES and non-AIBCES (Fig. 2 and Table 2).

**Table 2:** Comparison of 5-year survival rates taking disease stage into consideration

	AIBCES	Non-AIBCES	*p*
Number of patients	1343	792	
Time at risk (days)	1985,832	10,55,612	
Death	670 (50%)	455 (57%)	
5-year survival rate (%)			
All cStages	52.8%	45.70%	<0.01
cStage 0	87.5%	–	
cStage I	76.8%	74.3%	0.18
cStage II	55.1%	44.9%	<0.01
cStage III	38.0%	30.3%	<0.01
cStage IV	28.2%	18.3%	0.06

*AIBCES* Authorized Institute for Board Certified Esophageal Surgeon

Univariate analysis revealed that AIBCES vs. non-AIBCES is a significant factor for survival (HR 0.79), as are age (≥ 70 vs. < 70), sex (male vs. female), cT (T3–4 vs. Tis-2), cN (N1–3 vs. N0), cM (M1 vs. M0), clinical stages, and adjuvant therapies (Table 3). Multivariate Cox proportional hazard analysis stratified based on stages and adjuvant therapies (*n* = 2067) revealed that AIBCES vs. non-AIBCES is a significant factor (adjusted HR 0.80) for survival, as are age and sex (Table 4).

**Table 3:** Univariate Cox proportional hazard analysis of survival

Cox analyses	Univariable			
Factor	HR	*p*	95% CI	
Age ≧70 vs. < 70	1.45	< 0.001	1.28	1.64
Male vs. female	1.47	< 0.001	1.22	1.76
T3–4 vs. Tis-2 (*n* = 2066)^a^	2.28	< 0.001	2.01	2.57
N1–3 vs. N0 (*n* = 2076)^a^	2.09	< 0.001	1.85	2.37
M1 vs. M0 (*n* = 2077)	2.31	< 0.001	1.92	2.79
Clinical stage (UICC, *n* = 2069)^a^				
cStage 0	0.34	0.27	0.01	1.95
cStage I	ref			
cStage II	2.28	< 0.001	1.88	2.77
cStage III	3.72	< 0.001	3.08	4.52
cStage IV	5.31	< 0.001	4.15	6.78
AIBCES vs. non-AIBCES	0.79	< 0.001	0.70	0.89
Adjuvant therapy (*n* = 2133)^a^				
No radiation or chemotherapy	ref			
+Radiation therapy	5.09	< 0.001	3.56	7.09
+Chemotherapy	1.34	< 0.001	1.17	1.52
+Radiation and chemotherapy	2.33	< 0.001	1.95	2.77

*AIBCES* Authorized Institute for Board Certified Esophageal Surgeon

^a^Incidence rate ratios are shown due to proportional hazard violation

**Table 4:** Multivariate Cox proportional hazard analysis stratified by clinical stages and adjuvant therapies (*n* = 2067)

Factor	HR	*p*	95% CI	
Age ≧70 vs. < 70	1.46	< 0.001	1.29	1.66
Male vs. female	1.33	0.003	1.10	1.60
AIBCES vs. non-AIBCES	0.80	< 0.001	0.71	0.91

*AIBCES* Authorized Institute for Board Certified Esophageal Surgeon

## Discussion

This study demonstrated a significantly better 5-year survival rate among cStage II-III thoracic esophageal cancer patients treated with esophagectomy at an AIBCES compared to those treated at a non-AIBCES. Univariate and multivariate analyses stratified based on stages and adjuvant therapies revealed that the institute certification (AIBCES vs. non-AIBCES) is a significant independent factor for 5-year survival.

Esophagectomy remains the mainstay of potentially curative treatment for esophageal cancer. Despite recent advances in surgical techniques and perioperative management, esophagectomy remains a highly invasive procedure that brings a high degree of surgical stress to patients along with several serious and potentially fatal postoperative complications, including pneumonia, anastomotic leaks, and recurrent nerve palsy. Takeuchi et al. reported the 30-day mortality rate and operative mortality rate after esophagectomy to be 1.2% and 3.4%, respectively, based on information from a Japanese nationwide Web-based database (NCD) 2011 [7]. This mortality rate is higher than that for gastrectomy or hepatectomy in Japan [8, 9].

The Institute of Medicine Committee on Quality of Health Care in America reported the existence of a “quality chasm” that must be crossed to improve medical care [10]. They reported that there is a serious difference between the medical service that could be received and that which is actually received. They suggested that improvements in the effectiveness, equity, efficiency, responsiveness, financing, patient centeredness and timeliness of treatment, as well as drastic changes in the medical delivery system, were needed to resolve this problem [10]. Regarding esophagectomy and other complex cancer resections, several studies have demonstrated a strong association between operation volume (hospital volume) and outcomes such as mortality and postoperative complications [11–14]. This association between operation volume and surgical outcomes leads us toward specialized centers in, for example, England, Wales, Canada, and the Netherlands [15]. Munasinghe et al. examined the difference in in-hospital mortality after esophagectomy between England (centralized, median number of esophagectomy per year per hospital is 17.5) and the USA (not centralized, median number of esophagectomies per year per hospital is 2) [16]. They reported that the overall mortality is higher in the USA (5.5%) than in England (4.2%) and concluded that centralization of high-risk cancer surgery to centers of excellence with a high procedural volume translates to improved clinical outcomes. On the other hand, in Japan, Fujita et al. examined the respective numbers of esophagectomies per year to define low-volume and high-volume hospitals [17]. They concluded that high-volume hospitals with low 30-day mortality rates are defined as those where esophagectomy is performed 40 or more times per year. Using data from the NCD, Nishigori et al. recently reported that high-volume hospitals had lower risk-adjusted 30-day and operative mortality rates following esophagectomy than low-volume hospitals [12]. The unadjusted operative mortality rate in hospitals performing fewer than 10 procedures per year (5.1 per cent) was more than three times higher than that in hospitals performing 30 or more procedures annually (1.5%). Although the association between hospital volume and operative mortality rate in hospitals is evident, these reports focused on short-term outcomes. Few studies have examined survival outcomes between institutions certified by an authorized medical society and uncertified institutions at the national level using big data.

The relationship between surgical outcome and surgeon level is not always linear. For instance, hospital-specific differences in surgical mortality are not related to complication rates, but instead on the ability to avoid mortality from such complications; that is, high-volume esophagectomy centers are less likely to exhibit “failure to rescue.” [18]. Other aspects of care associated with improved outcomes include the ability of the care team, including nursing and staffing levels [19]. To improve not only short-term surgical outcomes (in-hospital mortality or surgical complications) but also total outcomes for esophageal cancer patients, the Japan Esophageal Society mandates that certified AIBCES are facilities able to provide surgeons with education and training toward becoming a board-certified esophageal surgeon who has the professional knowledge and skills needed to perform esophageal surgery. Because the aim of institutional certification is to contribute to the total improvement of national medical care in surgery for esophageal disease, the Japanese Esophageal Society examines not only the surgical achievements at an institution, but also achievements in research, diagnosis, and medical treatment and education.

It is not easy to verify the appropriateness of institutional certification by a medical society. For example, what is the most appropriate indicator with which to evaluate an institutional certification system? In 1966, Donabedian proposed a model of health-care quality that distinguished structure (the type and quantity of inputs used), process (the clinical procedures that use inputs to produce outputs), and outcomes (the type of quantity of outputs produced) [20]. These clinical indicators have been widely used for half a century. Among them, outcome appears to be most important indicator with which to verify the effectiveness of AIBCES certified by the Japan Esophageal Society. This is because outcome indicators (mortality or survival rate) depend not only on experienced surgeons, but also on a well-trained perioperative care team. In other words, it broadly reflects the ability of the institute.

There are three types of nationwide esophageal cancer registrations in Japan: (1) the National Database of Hospital-based Cancer Registries, (2) the NCD and (3) the site-specific registries managed by the Japan Esophageal Society. We will now consider what is the best data source to examine survival outcome and verify the effectiveness of institutional certification. The National Database of Hospital-based Cancer Registries is the first uniform registry system implemented nationwide in Japan for all types of cancer, including esophageal cancer. In 2007, the Ministry of Health, Labour and Welfare (Japan) designated 289 hospitals as Designated Cancer Care Hospitals throughout Japan to enhance cancer control activities. In addition, the Designated Cancer Care Hospitals also play a leading role collecting information on cancer care that will be entered in the Hospital-based Cancer Registries [6]. In 2015, however, a total of 702,866 cancer cases treated at 427 institutes were submitted. For esophageal cancer, 22,351 cancer cases treated at 426 institutes were submitted that year. The precise registry rules and definitions, which are covered by the tumor registrar training programs, are meant to ensure reliable data collection by non-physician tumor registrars. The data quality is ensured in three ways: (1) rigorous training of tumor registrars, (2) consistency-checking software, and (3) extensive support provided by the National Cancer Center staff. Moreover, in this registry, information on both clinical (c-) stages and pathologic (p-) stages is collected. The date of diagnosis is determined as the date when the most definitive diagnostic test was performed before treatment was prescribed.

On the other hand, the NCD was established in 2011 as a nationwide Web-based database in cooperation with the board certification system for surgery in Japan. It collected data on more than 1 million surgical cases from more than 4000 hospitals over a 1-year period. In 2011, 5354 patients at 713 hospitals underwent esophagectomy and were registered in the NCD. Thus, most esophagectomy cases performed in Japan appear to be captured by the NCD system. Although the NCD is the largest surgical database in Japan, it focuses on short-term surgical outcome and does not include survival data at this point. Because in the present study our focus was on long-term survival outcomes, we did not use the NCD registry. In 2008, 2439 patients who underwent esophagectomy along with their 5-year survival outcomes were registered in the Comprehensive Registry of Esophageal Cancer in Japan established by the Japan Esophageal Society. The median follow-up period was 41 (0–71) months, which is shorter than that of the National Database of Hospital-based Cancer Registries (53 (1–81) months). Among these patients, about 87% were registered as thoracic esophageal cancer. This number is nearly the same as the 2135 patients included in the Hospital-based Cancer Registries.

The present study has several limitations. First, only treatment information on first-course treatments is collected and provided by the registering facility. The term “first course” is defined as the set of standard treatments initially considered and subsequently administered at a facility for a given type of cancer and its stage. Treatments added after the start of therapy based on new findings or along the disease progression course are, by definition, not considered first course and are therefore not registered. For example, if definitive chemoradiotherapy was administered to a patient with Stage II thoracic esophageal cancer, and treatments induced complete remission at that time, the patient was registered as having esophageal cancer treated with chemoradiotherapy. When the main tumor re-grew and the patient underwent salvage esophagectomy, this surgical treatment was not registered according to the rule. It may be a limitation from the completeness of surgical data, but helps to set the inclusion criteria into our analysis. Second, the cancer data were collected only from Designated Cancer Care Hospitals, and the data are limited to facilities that had sufficient (≧ 90%) follow-up rate to avoid the potential bias caused by a differential censoring. As a result, the data did not cover all patients who underwent esophagectomy in Japan. Third, our data did not include the presence of comorbidity and performance status because the registry does not have the information. However, the age of patients between AIBCE and non-AIBCE were not different, potentially suggesting the similarity of comorbidity distribution between these two groups.

One interesting question remaining is whether the difference in survival between AIBCES and non-AIBCES is caused by certification itself or treatment volumes. Naturally, certified hospitals tend to have larger volume of patients than non-certified hospitals. Unfortunately, we could not obtain information on hospital volume in each institute for this study. Nonetheless, the difference is evident (25 per year at certified institutes and 5 per year at uncertified institutes). One provision of AIBCES is that more than 50 surgeries for esophageal disease must be performed within a period of 5 years, which suggests this hospital volume is sufficient to affect the result. As mentioned, however, a study using Japanese big data showed that more than 30–40 esophagectomies per year are necessary to improve surgical outcome, whereas the number of cases of esophagectomy needed for AIBCES certification is 10 per year. It thus appears that some other factor is more important than surgical volume for improving outcomes. A clue to that factor may be provided by our observation that for Stage I patients, there was no significant difference in 5-year survival between AIBCES and non-AIBCES, but there was a difference for cStage II–III patients. Tsukada et al. reported that patients at lower volume hospitals were less likely to receive neoadjuvant therapy for esophageal cancer [21]. To address that issue, we performed a multivariate Cox proportional hazard analysis stratified by adjuvant therapies, which revealed that AIBCES vs. non-AIBCES is a significant independent factor.

In conclusion, our findings indicate that the institute certification system used by the Japan Esophageal Society may be appropriate, as indicated by improved 5-year survival outcomes. This institute certification system has the potential to contribute to a more appropriate medical delivery system for thoracic esophageal cancer in the future.

## References

[1] Tachimori Y, Ozawa S, Numasaki H (2017). Registration Committee for Esophageal Cancer of the Japan Esophageal Society. Comprehensive Registry of Esophageal Cancer in Japan, 2010. Esophagus..

[2] Chen MF, Yang YH, Lai CH (2013). Outcome of patients with esophageal cancer: a nationwide analysis. Ann Surg Oncol.

[3] Tachimori Y, Ozawa S, Numasaki H (2016). Efficacy of lymph node dissection by node zones according to tumor location for esophageal squamous cell carcinoma. Esophagus.

[4] Ando N, Kato H, Igaki H (2012). A randomized trial comparing postoperative adjuvant chemotherapy with cisplatin and 5-fluorouracil versus preoperative chemotherapy for localized advanced squamous cell carcinoma of the thoracic esophagus (JCOG9907). Ann Surg Oncol.

[5] van Hagen P, Hulshof MC, van Lanschot JJ (2012). Preoperative chemoradiotherapy for esophageal or junctional cancer. N Engl J Med.

[6] Higashi T, Nakamura F, Shibata A (2014). The national database of hospital-based cancer registries: a nationwide infrastructure to support evidence-based cancer care and cancer control policy in Japan. Jpn J Clin Oncol.

[7] Takeuchi H, Miyata H, Gotoh M (2014). A risk model for esophagectomy using data of 5354 patients included in a Japanese nationwide web-based database. Ann Surg.

[8] Kunisaki C, Miyata H, Konno H (2017). Modeling preoperative risk factors for potentially lethal morbidities using a nationwide Japanese web-based database of patients undergoing distal gastrectomy for gastric cancer. Gastric Cancer..

[9] Takahara T, Wakabayashi G, Konno H (2016). Comparison of laparoscopic major hepatectomy with propensity score matched open cases from the National Clinical Database in Japan. J Hepatobiliary Pancreat Sci..

[10] Crossing the Quality Chasm: A New Health System for the 21st Century. Institute of Medicine (US) Committee on Quality of Health Care in America. Washington (DC): National Academies Press (US); 2001. 25057539

[11] Wouters MW, Gooiker GA, van Sandick JW (2012). The volume–outcome relation in the surgical treatment of esophageal cancer: a systematic review and meta-analysis. Cancer.

[12] Nishigori T, Miyata H, Okabe H (2016). Impact of hospital volume on risk-adjusted mortality following oesophagectomy in Japan. Br J Surg.

[13] Fuchs HF, Harnsberger CR, Broderick RC (2017). Mortality after esophagectomy is heavily impacted by center volume: retrospective analysis of the Nationwide Inpatient Sample. Surg Endosc.

[14] Markar SR, Mackenzie H, Askari A (2017). Effect of esophageal cancer surgeon volume on management and mortality from emergency upper gastrointestinal conditions: population-based cohort study. Ann Surg.

[15] Chang AC (2018). Centralizing esophagectomy to improve outcomes and enhance clinical research: invited expert review. Ann Thorac Surg..

[16] Munasinghe A, Markar SR, Mamidanna R (2015). Is it time to centralize high-risk cancer care in the United States? Comparison of outcomes of esophagectomy between England and the United States. Ann Surg.

[17] Fujita H, Ozawa S, Kuwano H (2010). Committee for Scientific Affairs, Japanese Association for Thoracic Surgery. Esophagectomy for cancer: clinical concerns support centralizing operations within the larger hospitals. Dis Esophagus..

[18] Ghaferi AA, Birkmeyer JD, Dimick JB (2011). Hospital volume and failure to rescue with high-risk surgery. Med Care.

[19] Silber JH, Rosenbaum PR, McHugh MD (2016). Comparison of the value of nursing work environments in hospitals across different levels of patient risk. JAMA surgery..

[20] Donabedian A (1966). Evaluating the quality of medical care. Milbank Mem Fund Q..

[21] Tsukada Y, Higashi T, Shimada H (2018). The use of neoadjuvant therapy for resectable locally advanced thoracic esophageal squamous cell carcinoma in an analysis of 5016 patients from 305 designated cancer care hospitals in Japan. Int J Clin Oncol..

